# Enzymatic Synthesis of High‐Density RNA Microarrays

**DOI:** 10.1002/cpz1.667

**Published:** 2023-02-16

**Authors:** Erika Schaudy, Jory Lietard, Mark M. Somoza

**Affiliations:** ^1^ Faculty of Chemistry, Institute of Inorganic Chemistry University of Vienna Josef‐Holaubek‐Platz 2 (UZA 2) Vienna Austria; ^2^ Chair of Food Chemistry and Molecular Sensory Science Technical University of Munich Lise‐Meitner‐Straße 34 Freising Germany; ^3^ Leibniz Institute for Food Systems Biology at the Technical University of Munich Lise‐Meitner‐Straße 30 Freising Germany

**Keywords:** microarray, RNA, T7 RNA polymerase

## Abstract

Oligonucleotide microarrays are used to investigate the interactome of nucleic acids. DNA microarrays are commercially available, whereas equivalent RNA microarrays are not. This protocol describes a method to convert DNA microarrays of any density and complexity into RNA microarrays using only readily available materials and reagents. This simple conversion protocol will facilitate the accessibility of RNA microarrays to a wide range of researchers. In addition to general considerations for the design of a template DNA microarray, this procedure describes the experimental steps of hybridization of an RNA primer to the immobilized DNA, followed by its covalent attachment via psoralen‐mediated photocrosslinking. The subsequent enzymatic processing steps comprise the extension of the primer with T7 RNA polymerase to generate complementary RNA, and finally the removal of the DNA template with TURBO DNase. Beyond the conversion process, we also describe approaches to detect the RNA product either by internal labeling with fluorescently labeled NTPs or via hybridization to the product strand, a step that can then be complemented by an RNase H assay to confirm the nature of the product. © 2023 The Authors. Current Protocols published by Wiley Periodicals LLC.

**Basic Protocol**: Conversion of a DNA microarray to an RNA microarray

**Alternate Protocol**: Detection of RNA via incorporation of Cy3‐UTP

**Support Protocol 1**: Detection of RNA via hybridization

**Support Protocol 2**: RNase H assay

## INTRODUCTION

RNA is essential in a variety of molecular processes, but the full spectrum of its interactions has not been fully explored. Platforms to investigate interactions of a vast number of different RNA molecules in parallel can be useful tools to deepen and massively accelerate our understanding of its diverse roles. Among such tools for the high‐throughput analysis of interactions of nucleic acids with proteins, small molecules, or other nucleic acids, such as DNA microarrays, have been proven particularly useful. These surfaces with immobilized oligonucleotide strands allow direct comparison of the impact of minor differences in DNA sequences on the interaction with other molecules, while enabling the identification of each variant via the assignment of a specific position on the surface. Applications of DNA microarrays are found in gene expression profiling (Hughes et al., 2001), in the investigation of binding interactions with transcription factors (Berger & Bulyk, 2009) and aptamers (Franssen‐van Hal et al., 2013; Katilius, Flores, & Woodbury, 2007), as well as in spatial transcriptomics (Salmén et al., 2018). Different production methods exist, yielding microarrays with varying densities, i.e., the number of variants per surface area. Although immobilization of pre‐synthesized oligonucleotides is a common method to produce DNA microarrays, a significantly higher density of spots can be achieved with *in situ* synthesis. The synthesis itself is based on the conventional phosphoramidite chemistry applied in the solid‐phase synthesis of DNA, and spatial resolution is achieved through inkjet printing, electrochemical deprotection, or photolithography. Although DNA microarrays are commercially available, equivalent RNA microarrays are not. This is particularly unfortunate considering the essential role of RNA and its interactions in different aspects of post‐transcriptional gene regulation, and our very limited knowledge on the RNA interactome. Over the years, different approaches have been explored to offer these platforms in RNA format. Progress has been slow, not least because of the intrinsic instability of RNA, making it more delicate to handle than DNA. Although the development of *in situ*‐synthesized DNA microarrays involves transferring and applying the principle of solid phase synthesis and phosphoramidite chemistry from beads to flat surfaces, the incompatibility of the common 2′‐OH deprotection strategies with glass surfaces explains why solid‐phase RNA synthesis is not immediately transferrable to microarrays. Synthesis of RNA microarrays therefore remains a challenge, with some success from several research groups on an experimental scale. Approaches based on spotted DNA libraries have been shown to yield surfaces with only low density (Phillips et al., 2018), whereas much higher densities can be achieved using very specialized infrastructure, for instance a re‐purposed Illumina sequencer (Ozer et al., 2015) or a setup for photolithographic microarray synthesis (Lackey, Mitra, Somoza, Cerrina, & Damha, 2009; Lietard, Ameur, Damha, & Somoza, 2018; Lietard, Damha, & Somoza, 2019; Lietard & Somoza, 2019; Wu, Holden, & Smith, 2014).

**Figure 1:**
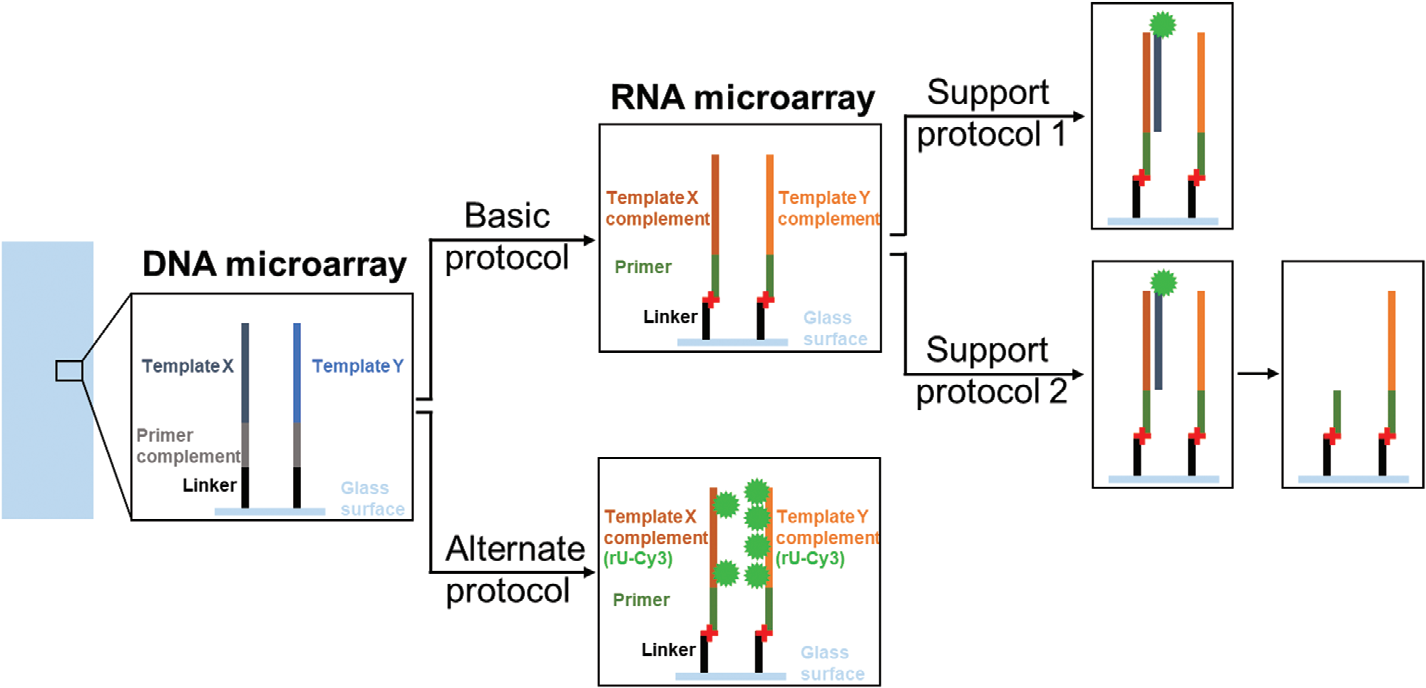
Flowchart of the protocols described in this manuscript.

The method we present herein is based on the enzymatic conversion of a DNA microarray via T7 RNA polymerase‐mediated primer extension, followed by DNase degradation of the template to yield strands of single‐stranded RNA spatially organized identically to the original DNA strands. Whereas these aspects are similar to the approach previously described by Wu et al., UV light–induced immobilization of the primer ensures higher flexibility in that it enables the transfer of the principle to DNA microarrays from commercial sources. The various protocols and the expected results are schematically summarized in Figure 1. The Basic Protocol describes the covalent binding of the primer, its enzymatic extension, and the DNA template degradation with TURBO DNase to generate an RNA microarray. The Alternate Protocol describes the primer extension in presence of Cy3‐UTP for incorporation of a fluorescently labeled nucleotide, yielding labeled RNA. The Support Protocols provide instructions for detecting RNA via hybridization with a fluorescently labeled complementary DNA probe, and for performing a subsequent RNase H assay to verify the nature of the product.

*CAUTION*: UV safety glasses should be worn when using UV light for photocrosslinking.

## STRATEGIC PLANNING

### Criteria for the Design of a DNA Template Microarray

**Figure 2:**
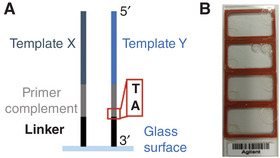
Theoretical considerations in the design of a DNA template microarray. **(A)** General structure of DNA template strands suitable for conversion to RNA. **(B)** A 4 × 44 K DNA microarray from Agilent with custom hybridization chambers (Grace Bio‐Labs RD500958). The solution for hybridization is added only to one of the chambers (second from the top), leaving an air bubble for rotational mixing. Chambers are also used for the other three arrays to protect them from mechanical damage during handling.

In general, the sequences on the DNA template microarray will depend on the intended application of the RNA microarray. However, as the conversion method is based on immobilized DNA oligonucleotides with the structure —glass‐3′‐Linker‐Primer_complement‐Template‐5′ (see Fig. 2), we recommend the following considerations in the design of the DNA template array.

The conditions presented herein are optimized for the use of the 15mer 2′*O*‐methyl RNA primer UAGGGACACGGCGAA with a 5′ terminal psoralen‐C2 modification. To prevent hybridization and crosslinking of the primer at unintended positions, we recommend consideration of its potential for cross‐hybridization with all template sequences during the design process.

The position of the interstrand crosslink is crucial for successful primer extension. Psoralen‐mediated photocrosslinking is based on psoralen intercalation followed by the cycloaddition reaction with a nearby pyrimidine. The highest efficiency for this reaction has been described for crosslinking to a thymine with a 3′ adjacent adenosine. We therefore recommend keeping this specific template strand sequence pattern (5′‐TA‐3′) at the photocrosslinking site.

We also advise the implementation of internal control sequences. Hybridization to specific RNA sequences with a fluorescently labeled oligonucleotide (see Support Protocol 1) can be used to assess the success of the conversion process. This detection method will leave RNA strands to be used in downstream experiments unaffected, provided that the control sequences selected do not to allow hybridization to any sequences of interest. This strategy even allows for the specific degradation of the RNA control with RNase H, whereas the other product strands remain intact (see Support Protocol 2). If an RNase H assay is carried out to confirm RNA conversion, select an RNA oligonucleotide that is not needed in any downstream application as a target for this hybridization to prevent the loss of this particular RNA sequence.

The length of the linker affects the accessibility of the immobilized nucleic acid strands by enzymes, and it should be inert, i.e., its sequence should not interact with any sequence of interest. Although non‐nucleosidic linkers appear to be an appropriate solution, some manufacturing processes of commercial microarrays are restricted to the incorporation of DNA nucleotides. Therefore, we investigated DNA homopolymer linkers, and our results show the highest yield of RNA with a T_10_ linker.

Several suppliers offer customizable DNA microarrays. Different restrictions regarding oligonucleotide length, spot size, and density apply due to differences in manufacturing processes, which have to be considered in the design operation. Several microarray variants with varying distributions of features on the surface are available, such as 4 × 44 K or 2 × 105 K with four or two individual arrays on a single slide, respectively. Although increasing the surface area per array allows for the accommodation of more sequence variants, such a setup also requires larger hybridization chambers to address each array individually. This can affect the efficiency of rotational mixing and can potentially lead to incomplete removal of solutions due to impaired accessibility to the chamber contents. The size of the array will also direct the dimensions of the hybridization chamber to be used.

## CONVERSION OF A DNA MICROARRAY TO AN RNA MICROARRAY

**Figure 3:**
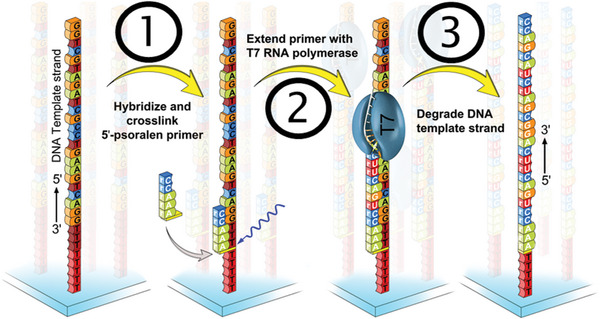
Schematic overview of the major processing steps in the conversion of a DNA template microarray to an RNA microarray.

The Basic Protocol guides the reader through the three essential steps required to convert a DNA microarray into an RNA microarray, as shown in Figure 3. After incubation with an excess of the primer probe for hybridization and stringency washes to minimize unspecific interactions and remove the unbound probe, a covalent bond in the form of an interstrand crosslink is established between the C2‐psoralen modification on the 5′ terminus of the primer and the DNA linker via exposure to UV light at 365 nm (step 1). Unreacted primer is removed with water, followed by pre‐conditioning of the array surface as a preparation for the first enzymatic processing namely, primer extension with T7 RNA polymerase (step 2). Step 3 consists of the degradation of the DNA template with TURBO DNase.

The method described herein has been developed on DNA microarrays produced via photolithographic synthesis (Hölz, Lietard, & Somoza, 2017; Lietard et al., 2021; Lietard, Schaudy, Hölz, Ameur, & Somoza, 2019; Sack, Kretschy, Rohm, Somoza, & Somoza, 2013). The design of a customized commercial DNA microarray from Agilent is based on insights from this development process. The volumes of solutions to prepare depend on the layout of the microarray and the corresponding type of hybridization chamber used. For illustrative purposes, this protocol describes all experimental details for two types of hybridization chambers, adapted to either photolithographic microarrays (type SA200) or commercial 4 × 44 K Agilent microarrays (type RD500958). Nonetheless, we mention all reagent concentrations so that volumes can be adapted to any type of hybridization chamber.

### Materials

Non‐stringent washing buffer (NSWB; composition see below)
Stringent washing buffer (SWB; composition see below).
Final washing buffer (FWB; 0.1× SSC)
RNase‐free water (Carl Roth, art. no. T143.5)
RNase AWAY surface decontaminant (ThermoFisher Scientific, cat. no. 7002)
Acetylated BSA (10 mg/ml) (Promega, cat. no. R3961)
Primer oligonucleotide (2′*O*‐methyl RNA with 5′ psoralen‐C2 modification: 5′‐Ps‐UAGGGACACGGCGAA, 1 µM in RNase‐free water) (Eurogentec)
2× MES hybridization buffer (composition see below)
Triton X‐100 (Sigma Aldrich, cat. no. T8787)
DTT (0.1 M) (e.g., ThermoFisher Scientific, cat. no. 707265ML)
RNase inhibitor, human placenta (40 U/µl) (NEB, cat. no. M0307)
NTP set (100 mM each) (ThermoFisher Scientific, cat. no. R0481)

**Figure 4:**
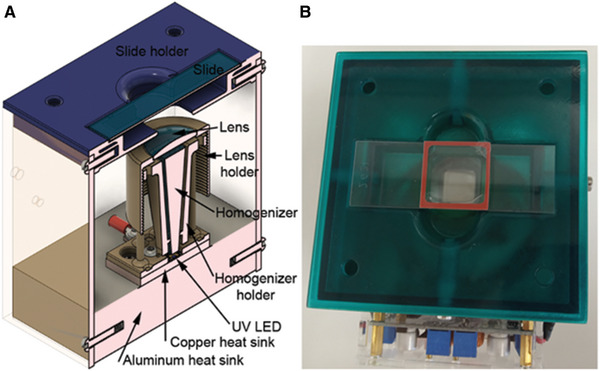
Custom‐made setup for photocrosslinking. **(A)** Rendering of the device. **(B)** Photograph of a slide with an SA200 hybridization chamber positioned for photocrosslinking. (Images reproduced from Schaudy et al. (2022) under CC BY 4.0.).

NOTE: We recommend storing the reaction buffers and the acetylated BSA in smaller aliquots to avoid repeated freeze‐thaw cycles and allow for faster thawing. Keep enzymes on ice until use.

Skirted reagent tubes with screw cap, 50 ml (e.g., Greiner bio‐one, VWR 525‐0396)
Microcentrifuge tubes Biopur Safe‐Lock 1.5 ml (VWR 211‐2161)
Micropipettes
Sterile filter tips (Starlab S1121‐3810, S1120‐1840, and S1126‐7810)
Hybridization oven (e.g., Boekel Scientific “Little Shot” 230500)
DNA template microarray
Self‐adhesive hybridization chambers, whose dimensions are adapted to the layout of the microarray (e.g., Grace Bio‐Labs SecureSeal SA200, RD500958 for Agilent SurePrint 4 × 44 K DNA microarray)
Tweezers
Adhesive seal tabs (Grace Bio‐Labs ST200)
Aluminum foil
Timer
Slide spinner (Labnet International C1303‐T)
Fridge or cold room at ∼4°C
High‐power Nichia NVSU333A 365 nm UV LED in a custom‐made setup for photocrosslinking, as previously described in detail (Schaudy, Hölz, Lietard, & Somoza, 2022) and shown in Figure 4
SÜSS Model 1000 UV intensity meter with a 365‐nm probe (alternative: Ushio UIT 201 Digital UV intensity meter with Ushio UVD‐365PD 365‐nm probe)

### Preparation of reagents and workplace

1 Prepare aliquots of the washing buffers NSWB and SWB (∼30 ml each) in 50‐ml reagent tubes.
2 Prepare an aliquot of 1× FWB (∼30 ml).
  Mix 3 ml of FWB 10× with 27 ml of RNase‐free water in a 50‐ml reagent tube.
3 Store the aliquots at room temperature in the dark until use.
4 Clean all surfaces and pipettes with RNase AWAY.

### Hybridization of the primer

5 Prepare an RNase‐free 1.5‐ml microcentrifuge tube.
6 Let acetylated BSA and primer oligonucleotide (1 µM) thaw at room temperature.
7 Switch on the hybridization oven and set the temperature to 37°C.
  If another primer is used, the hybridization temperature should be adjusted in accordance with its melting temperature.
8 Prepare the hybridization solution by mixing the reagents according to Table 1 in a sterile 1.5‐ml microcentrifuge tube.

**Table 1:** Reaction Mixture for Hybridization of the Primer

			Hybridization chamber type	
	Stock conc.	Final conc.	RD500958	SA200
MES hybridization buffer	2 ×	1 ×	117.50 µl	140.00 µl
Acetylated BSA	10 mg/ml	0.44 mg/ml	10.42 µl	12.41 µl
Primer oligonucleotide	1 µM	0.089 µM	20.92 µl	24.92 µl
RNase‐free water			86.17 µl	102.67 µl
Total volume			235.00 µl	280.00 µl

9 Place the glass slide with the DNA template microarray on the bench.
  Make sure the side with DNA is facing up.
10 Take a hybridization chamber of the correct size, use tweezers to remove the thin plastic foil, and expose the adhesive on the rubber gasket.
11 Place the hybridization chamber over the DNA array, stick it to the glass surface, and then fix it by tightly pressing on the rubber gasket.
  See “Troubleshooting” for help with positioning.
12 Mix the hybridization solution by flipping the tube carefully.
  The hybridization buffer contains Tween 20, resulting in bubble formation upon excessive mixing. The presence of bubbles can complicate the application of the solution in the following step.
13 Carefully add the hybridization solution to the chamber with a pipette through one of the holes of the hybridization chamber.
  Dispense by pressing the plunger to the first stop, then slowly release residual solution without applying air, and keep the plunger pressed until the tip is displaced from the hybridization chamber.
14 Seal the holes with adhesive seal tabs.
15 Briefly rotate the slide to ensure proper mixing is facilitated by movement of the air bubble.
16 Wrap the slide with aluminum foil.
17 Place the slide in the hybridization oven and incubate it for 30 min at 37°C with rotation.

### Stringency washes

18 Carefully remove the adhesive seal tabs.
19 Pipette out the hybridization solution and discard it.
20 Remove the hybridization chamber with tweezers.
21 Place the slide in the 50‐ml tube with 30 ml of NSWB and shake vigorously for 1 min.
22 Rinse off residual buffer on the walls of the reagent tube to prevent carry‐over into the next solution.
23 Place the slide in the 50‐ml reagent tube with 30 ml of SWB and shake vigorously for 30 s.
24 Repeat step 22.
25 Place the slide in the 50‐ml reagent tube with 30 ml of 1× FWB and shake for ∼5 s.
  Make sure that no salt deposit is left on the array.
26 Place the slide in a microarray centrifuge and spin dry for ∼30 s.
  At this point, the slide can either be stored in a desiccator until use, or the next steps of the procedure can be carried out right away.

### Crosslinking

27 Place the UV light source at 4°C for approximately 30 min to equilibrate the temperature of the whole device.
28 Measure the UV light intensity with an intensity meter over a period of 60 s; note both the initial and final intensities, and then calculate the average of these two values.
29 Calculate the exposure time required to achieve a radiant exposure of 25 J/cm² based on the average intensity value calculated in step 28.
  Use the following formula: exposure time [s] = radiant exposure [J/cm²] / intensity [W/cm²]
30 Prepare 1 volume of 1× MES hybridization buffer in a sterile 1.5‐ml microcentrifuge tube.
  Mix 2× MES hybridization buffer 1:1 with RNase‐free water. Prepare a sufficient amount to fill the chamber completely (Table 2).

**Table 2:** Solution for Crosslinking

			Hybridization chamber type	
	Stock conc.	Final conc.	RD500958	SA200
MES hybridization buffer	2 ×	1 ×	137.50 µl	175.00 µl
RNase‐free water			137.50 µl	175.00µl
Total volume			275.00 µl	350.00 µl

31 Place the slide on the bench with the DNA array facing up.
32 Take a hybridization chamber of the correct size and remove the thin plastic foil to expose the adhesive on the rubber gasket.
33 Place the hybridization chamber over the DNA array, stick it to the glass surface, and then fix it by tightly pressing on the rubber gasket.
34 Apply 1× MES buffer to completely fill the reaction chamber.
  To avoid overfilling, watch the second hole when applying the solution. If the solution is stuck between the glass slide and the plastic cover, tilt it while filling.
35 Seal the holes of the reaction chamber with adhesive seal tabs.
36 Place the glass slide above the UV light source with the hybridization chamber facing upward.
37 Adjust its position to be well in front of the UV light.
  See Figure 4B for an example of correct positioning.
38 Plug in the power supply to start UV light exposure and set a timer to the exposure time calculated in 29.
39 Prepare the pre‐incubation solution (Table 3).

**Table 3:** Pre‐Incubation Mixture

			Hybridization chamber type	
	Stock conc.	Final conc.	RD500958	SA200
RNAPol reaction buffer	10 ×	1 ×	23.5 µl	28 µl
Triton X‐100	0.25 %	0.005 %	4.7 µl	5.6 µl
Acetylated BSA	10 mg/ml	0.2 mg/ml	4.7 µl	5.6 µl
RNase‐free water			202.1 µl	240.8 µl
Total volume			235.00 µl	280.00 µl

40 Unplug the power supply to switch off the UV light and take the slide to the bench.
41 Carefully remove the adhesive seal tabs from the hybridization chamber with tweezers.
  The hybridization chamber should stay attached to the glass slide.
42 Pipette out the buffer and discard it.
43 Pipette 1 volume of RNase‐free water in and out of the hybridization chamber for 30 s, and then discard the water.
44 Repeat step 43 for another 30 s.

### Pre‐incubation

Pre‐incubation serves to equilibrate the surface, remove potential leftovers of EDTA, which would result in inhibition of the enzymatic reactions due to complexation of the required cofactor, and prevent adhesion of the polymerase to the surface by blocking it with acetylated BSA.

45 Apply the pre‐incubation solution to the hybridization chamber.
46 Seal the holes with adhesive seal tabs.
47 Wrap the slide in aluminum foil.
48 Incubate at 37°C for ∼30 min with rotation in the hybridization oven.
49 In the meantime, prepare the primer extension reaction solution (Table 4)—except for the polymerase—in a sterile microcentrifuge tube at room temperature.

**Table 4:** Primer Extension Reaction Mixture

			Hybridization chamber type	
	Stock conc.	Final conc.	RD500958	SA200
RNAPol reaction buffer	10 ×	1 ×	23.5 µl	28 µl
Triton X‐100	0.25 %	0.005 %	4.7 µl	5.6 µl
DTT	100 mM	4 mM	9.4 µl	11.2 µl
RNase inhibitor, human placenta	40 U/µl	1 U/µl	5.88 µl	7 µl
NTP mix	2.5 mM each	0.5 mM each	47 µl	56 µl
T7 RNA polymerase	50 U/µl	3 U/µl	14.1 µl	16.8 µl
RNase‐free water			130.4 µl	155.4 µl
Total volume			235.00 µl	280.00 µl

50 Remove the adhesive seal tabs from the hybridization chamber.
51 Pipette out the solution and discard it.

### Primer extension

52 Add T7 RNA polymerase to the primer extension reaction solution.
53 Mix by carefully pipetting up and down.
54 Apply the solution to the hybridization chamber.
55 Seal the holes with adhesive seal tabs and test for movement of the solution by rotating the slide manually.
56 Wrap the slide in aluminum foil.
57 Incubate at 37°C with rotation for 16 hr (overnight) in the hybridization oven.
58 At the end of the incubation time, prepare the reaction mix for the DNA template degradation (Table 5)—except for the DNase—in a sterile microcentrifuge tube at room temperature.

**Table 5:** Reaction Mixture for DNA Template Degradation

			Hybridization chamber type	
	Stock conc.	Final conc.	RD500958	SA200
TURBO DNase reaction buffer	10 ×	1 ×	23.50 µl	28.00 µl
RNase inhibitor, human placenta	40 U/µl	1 U/µl	5.88 µl	7.00 µl
TURBO DNase	2 U/µl	0.1 U/µl	11.75 µl	14.00 µl
RNase‐free water			193.90 µl	231.00 µl
Total volume			235.00 µl	280.00 µl

59 Remove the adhesive seal tabs.
60 Pipette out the solution and discard it.

### TURBO DNase reaction

61 Add TURBO DNase to the reaction mixture and mix it by carefully pipetting up and down.
62 Apply the reaction mixture for DNA template degradation to the hybridization chamber.
63 Seal the holes with adhesive seal tabs.
64 Wrap the slide in aluminum foil.
65 Incubate at 37°C with rotation for 5 hr in the hybridization oven.
66 Remove the adhesive seal tabs.
67 Pipette out the solution and discard it.
68 Take 1 chamber volume of NSWB buffer, pipette it in and out of the chamber, and then discard the solution.
69 Repeat step 68.
70 Remove the hybridization chamber with tweezers and discard it.
71 Place the slide in a reagent tube with ∼30 ml of 1× FWB.
72 Shake for 5 s.
73 Place the slide in a microarray centrifuge and spin dry for ∼30 s.
  At this point, the slide can either be stored in dry conditions in a desiccator until further use or used directly, e.g., to detect RNA via hybridization, as described in Support Protocol 1.

## DETECTION OF RNA VIA INCORPORATION OF Cy3‐UTP

The Basic Protocol yields an array with single‐stranded RNA. For direct detection of the RNA product, the primer extension reaction mixture can be supplemented with Cy3‐UTP to achieve internal labeling. As this labeling will affect all RNA sequences containing a “U,” the potential effects of the presence of a fluorescent dye on downstream applications of the RNA microarray should be taken into consideration.

### Additional Materials (also see Basic Protocol)

Cy3‐UTP (1 mM) (Jena Bioscience, cat. no. NU‐821‐CY3)
Microarray scanner, e.g., GenePix 4400A (Molecular Devices) with laser and emission filter for the fluorescent dye used, e.g., emission filter 575DF35 (558 nm to 593 nm) for the green laser to detect Cy3

For direct labeling of the RNA through incorporation of a fluorescent nucleotide, follow Basic Protocol until step 73, except for a change in preparation of the mix for enzymatic primer extension in step 49, which should be changed to include Cy3‐UTP according to Table 6. After washing and drying following enzymatic degradation of the DNA template (step 73), the slide can directly be scanned with a microarray scanner at a wavelength of 532 nm.

**Figure 5:**
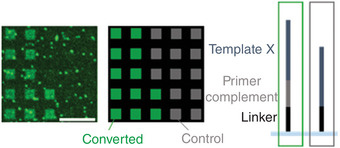
Excerpt of an illustrative microarray scan after primer extension in presence of Cy3‐UTP. “Converted” and “control” sequences only differ in the presence of a sequence complementary to the primer on the initial DNA template microarray (scale bar: 100 µm).

An example of an expected result of such a scan is shown in Figure 5.

**Table 6:** Reaction Mix for Primer Extension With Internal Labeling via Incorporation of Cy3‐UTP

			Hybridization chamber type	
	Stock conc.	Final conc.	RD500958	SA200
RNAPol reaction buffer	10 ×	1 ×	23.50 µl	28.00 µl
Triton X‐100	0.25 %	0.005 %	4.70 µl	5.60 µl
DTT	100 mM	4 mM	9.40 µl	11.20 µl
RNase inhibitor, human placenta	40 U/µl	1 U/µl	5.88 µl	7.00 µl
ATP	10 mM	0.5 mM	11.75 µl	14.00 µl
CTP	10 mM	0.5 mM	11.75 µl	14.00 µl
GTP	10 mM	0.5 mM	11.75 µl	14.00 µl
UTP	10 mM	0.2 mM	4.70 µl	5.60 µl
Cy3‐UTP	0.5 mM	0.0175 mM	8.23 µl	9.80 µl
T7 RNA polymerase	50 U/µl	3 U/µl	14.10 µl	16.80 µl
RNase‐free water			130.40 µl	155.40 µl
Total volume			235.00 µl	280.00 µl

## Support Protocol 1 DETECTION OF RNA VIA HYBRIDIZATION

A fluorescently labeled probe can be hybridized to the single‐stranded RNA as the product of the conversion process described in the Basic Protocol. In contrast to internal labeling (see Alternate Protocol), this approach allows to detect the presence of RNA strands on the array in a sequence‐specific manner. Performed as an additional step following the Basic Protocol, the complementary RNA will be the only target of hybridization. We recommend including a designated control sequence in the design (see Strategic Planning) to leave the RNA strands required in downstream experiments unaffected by these processing steps. The signal intensity detected after carrying out this protocol can serve as evidence for successful conversion to RNA.

If an RNase H assay (see Support Protocol 2) is planned as a consecutive step, a DNA probe should be used for hybridization. As RNase H will degrade the hybridized RNA strand, consider targeting an RNA oligonucleotide that is not needed in any downstream application and pay attention to potential cross‐hybridization as well.

### Additional Materials (also see Basic Protocol)

Fluorescently labeled (e.g., Cyanine3) DNA probe with the sequence identical to the template sequence intended as control (0.1 µM, e.g., from Eurogentec) [For example, the product of conversion of the template 5′‐TCAACCCAGGTCCAATTTCC‐3′ can be detected with the probe 5′‐Cy3‐TCAACCCAGGTCCAATTTCC‐3′)]
Microarray scanner, e.g., GenePix 4400A (Molecular Devices) with laser and emission filter for the fluorescent dye used, e.g., emission filter 575DF35 (558 nm to 593 nm) for the green laser to detect Cy3

### Preparation

1 Prepare aliquots of the washing buffers NSWB and SWB (∼30 ml each) in 50‐ml reagent tubes.
2 Prepare an aliquot of 1× FWB (∼30 ml).
  Mix 3 ml of FWB 10× with 27 ml of RNase‐free water in a 50‐ml reagent tube.
3 Store the aliquots at room temperature in the dark until use.
4 Clean all surfaces and pipettes with RNase AWAY.
5 Switch on the hybridization oven and set the temperature to 37°C.
  The hybridization temperature should be adjusted according to the melting temperature of the probe.

### Hybridization with a fluorescently labeled probe

6 Let acetylated BSA and the Cy3‐labeled complementary oligonucleotide (0.1 µM) thaw at room temperature in the dark.
7 Prepare the hybridization solution by mixing the reagents according to Table 7 in a sterile 1.5‐ml microcentrifuge tube.
  Avoid extensive light exposure of solutions containing reagents with fluorescent dyes.

**Table 7:** Solution for Hybridization With a Fluorescently Labeled Complement

			Hybridization chamber type	
	Stock conc.	Final conc.	RD500958	SA200
MES hybridization buffer	2 ×	1 ×	117.50 µl	140.00 µl
Acetylated BSA	10 mg/ml	0.44 mg/ml	10.42 µl	12.41 µl
Cy3‐labeled DNA probe	100 nM	12.2 nM	28.75 µl	34.25 µl
RNase inhibitor, human placenta	40 U/µl	0.07 U/µl	0.39 µl	0.47 µl
RNase‐free water			77.94 µl	92.87 µl
Total volume			235.00 µl	280.00 µl

8 Place the slide on the bench with the RNA facing up.
9 Take a hybridization chamber of the correct size and remove the thin plastic foil to expose the adhesive on the rubber gasket.
10 Place the hybridization chamber over the RNA array, stick it to the glass surface, and then fix it by tightly pressing on the rubber gasket.
11 Mix the hybridization solution by flipping the tube.
  The hybridization buffer contains Tween 20, resulting in bubble formation upon excessive mixing. The presence of bubbles can complicate the application of the solution in the following step.
12 Carefully add the hybridization solution to the chamber with a pipette through one of the holes of the hybridization chamber.
  Dispense by pressing the plunger to the first stop, then slowly release residual solution without applying air, and keep the plunger pressed until the tip is displaced from the hybridization chamber.
13 Seal the holes with adhesive seal tabs.
14 Briefly rotate the slide to ensure proper mixing is facilitated by movement of the air bubble.
15 Wrap the slide with aluminum foil.
16 Place the slide in the hybridization oven and incubate for 60 min at 37°C with rotation.

### Stringency washes and scanning

**Figure 6:**
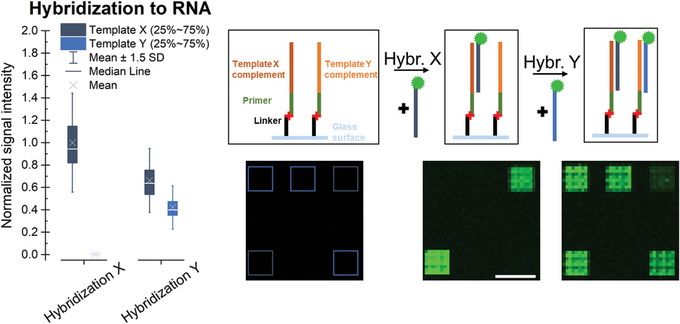
Detection of RNA via hybridization. Upon hybridization with a fluorescently labeled probe X, only features with complementary RNA, shown in the scheme with dark blue boxes, yield a signal. RNA of another sequence (template complement Y, light blue boxes in the scheme) can be detected in a subsequent hybridization with probe Y (scale bar: 100 µm).

17 Carefully remove the hybridization chamber with tweezers.
18 Place the slide in a 50‐ml reagent tube with 30 ml of NSWB and shake vigorously for 2 min.
19 Strip off residual buffer on the walls of the reagent tube to prevent rigorous carry‐over to the next solution.
20 Place the slide in a 50‐ml reagent tube with 30 ml of SWB and shake vigorously for 1 min.
21 Repeat step 19.
22 Place the slide in a 50‐ml reagent tube with 30 ml of 1× FWB and shake for 10 s.
  Make sure that no salt deposit is left on the array.
23 Place the slide in a microarray centrifuge and spin dry for ∼30 s.
24 Scan the slide using a microarray scanner.
  For detecting the Cyanine3 signal, set the excitation wavelength to 532 nm and use an emission filter for the green laser (558 nm to 593 nm; 575DF35). For detecting other dyes, adjust the settings of the scanner accordingly.
  An example of RNA strand detection by hybridization with Cy3‐labeled probes is shown in Figure 6.

## Support Protocol 2 RNase H ASSAY

**Figure 7:**
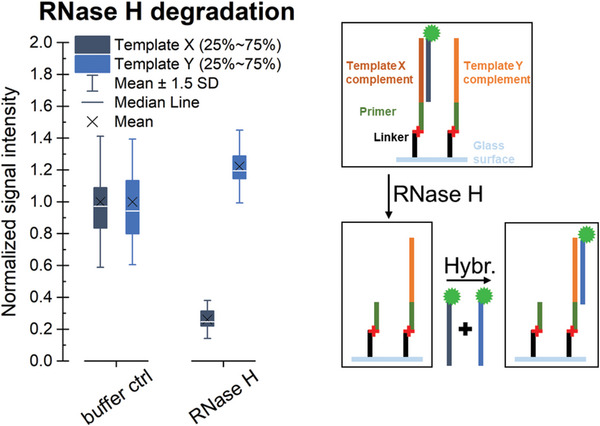
Effect of RNase H degradation on RNA in an RNA:DNA duplex versus single‐stranded RNA can be monitored via hybridization.

**Figure 8:**
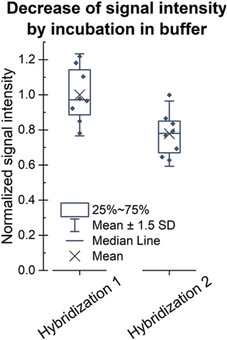
Incubation in buffer results in some loss of RNA from the surface.

To verify that the product of the conversion is RNA, the array can be treated with RNase H, which specifically degrades RNA in duplex with DNA. Therefore, RNA strands not targeted by the hybridization with a fluorescently labeled DNA probe are not affected by the treatment (compare Fig. 7). However, since even incubation in buffer in absence of the RNase results in loss of RNA from the surface (Fig. 8), the execution of this protocol should be envisaged only if there is a need to verify the identity of the polymerized strand. As this protocol requires the presence of an RNA:DNA duplex, we recommend following the Basic Protocol and refrain from internal labeling (described in the Alternate Protocol), but use hybridization with a fluorescently labeled DNA probe as a detection method.

Be aware that at this point, the template DNA is already degraded. RNase H treatment will degrade only the RNA complementary to the fluorescently labeled oligonucleotide probe used for hybridization‐based detection. The respective RNA strands cannot be reconstructed due to the absence of a DNA template.

### Additional Materials (also see Basic Protocol)

Fluorescently (e.g., Cyanine3) labeled DNA probe with the sequence identical to the template sequence intended as control (0.1 µM, e.g., from Eurogentec; for example, the product of conversion of the template 5′‐TCAACCCAGGTCCAATTTCC‐3′ can be detected with the probe 5′‐Cy3‐TCAACCCAGGTCCAATTTCC‐3′)
Microarray scanner e.g., GenePix 4400A (Molecular Devices) with laser and emission filter for the fluorescent dye used, e.g., emission filter 575DF35 (558 nm to 593 nm) for the green laser to detect Cy3)
RNase H (5 U/µl) (NEB, cat. no. M0297) supplemented with RNase H reaction buffer 10×

### Hybridization with DNA

1 Follow all steps outlined in Support Protocol 1 for hybridization with a fluorescently labeled DNA probe.

### RNase H assay

2 Prepare the reaction mixture for the RNase H degradation according to Table 8.
  We recommend handling RNase H in a designated area in the laboratory and using a specific set of buffers to avoid contamination of other experiments that involve RNA. After the experiment, solutions containing RNase H are collected in a microcentrifuge tube, which is closed and discarded.

**Table 8:** Reaction Mixture for RNase H Assay

			Hybridization chamber type	
	Stock conc.	Final conc.	RD500958	SA200
RNase H reaction buffer	10 ×	1 ×	23.5 µl	28 µl
RNase H	5 U/µl	0.02 U/µl	0.94 µl	1.12 µl
RNase‐free water			210.6 µl	250.9 µl
Total volume			235.00 µl	280.00 µl

3 Place the slide on the bench with the RNA facing upward.
4 Take a hybridization chamber of the correct size and remove the thin plastic foil to expose the adhesive on the rubber gasket.
5 Place the hybridization chamber over the array, stick it to the glass surface, and then fix it by tightly pressing on the rubber gasket.
6 Mix the solution by pipetting up and down.
7 Apply the reaction mix to the hybridization chamber.
8 Seal the holes with adhesive seal tabs.
9 Wrap the slide in aluminum foil.
10 Incubate at 37°C with rotation for 1 hr in the hybridization oven.

### Washing

11 Remove the adhesive seal tabs.
12 Pipette the solution out of the hybridization chamber and into a microcentrifuge tube, and then close and discard it.
13 Take 1 chamber volume of NSWB buffer and pipette it in and out of the chamber, collect it in a microcentrifuge tube, which is then closed and discarded.
14 Repeat step 13.
15 Remove the hybridization chamber with tweezers and discard it.
16 Place the slide in a 50‐ml reagent tube with ∼30 ml of 1× FWB.
17 Shake for 5 s.
18 Dry the slide in a microarray centrifuge.
19 Discard the 1× FWB used for washing.
20 Scan the slide using a microarray scanner to detect residual signal as a control.
  For detecting the Cyanine3 signal, set the excitation wavelength to 532 nm and use an emission filter for the green laser (558 nm to 593 nm; 575DF35). For detecting other dyes, adjust the settings of the scanner accordingly.

### Hybridization with DNA

21. To verify the effect of RNase H degradation, repeat step 1 and compare the fluorescent signal intensities between the two hybridization steps.

Additionally, hybridization to a second RNA control sequence can be performed according to Support Protocol 1 to verify its integrity after the RNase H assay (compare Fig. 7).

Partial loss of RNA due to incubation in an aqueous buffer should be taken into account, as illustrated in Figure 8.

## REAGENTS AND SOLUTIONS

### 12× MES stock buffer (1.22 M MES, 0.89 M Na^+^) (500 ml total volume)

35.2 g MES free acid monohydrate (Carl Roth, cat. no. 6066.1)
96.65 g MES sodium salt (Carl Roth, cat. no. 6922.2)
Milli‐Q water (not DEPC‐treated) to 500 ml

Store protected from light at 4°C for up to 24 months

Dissolve the salts in water and then bring to a 500‐ml final volume. Check and adjust the pH to 6.5‐6.7, and then filter through a sterile filter unit with 2‐µm pore size (VWR 514‐0332).

### 2× MES hybridization buffer [200 mM MES, 1 M Na^+^, 20 mM EDTA, 0.01% (v/v) Tween 20)] (250 ml total volume)

41.5 ml of 12× MES stock buffer
88.5 ml of 5 M NaCl (Sigma Aldrich S5150)
20 ml of 0.5 M EDTA (Sigma Aldrich E7889)
0.5 ml of 10% Tween 20 (Sigma Aldrich 11332465001)
99.5 ml of Milli‐Q water (not DEPC‐treated)

Store protected from light at 4°C for up to 24 months

Mix the components in a measuring cylinder, and then filter through a sterile filter unit with 2‐µm pore size (VWR 514‐0330).

### Cy3‐UTP solution (0.5 mM)

Cy3‐UTP (1 mM) (Jena Bioscience, cat. no. NU‐821‐CY3)
RNase‐free water (Carl Roth, art. no. T143.5)

Store aliquots at −20°C and avoid repeated freeze–thaw cycles. Keep in the dark to avoid extensive light exposure of the fluorescent dye.

Mix 1:1 water and Cy3‐UTP (1 mM)

### Final washing buffer, 10× FWB (0.1× SSC) (250 ml total volume)

12.5 ml of 20× SSC (Carl Roth, cat. no. 1054.1)
237.5 ml of Milli‐Q water (not DEPC‐treated)

Store at 4°C for up to 24 months.

Mix the components in a measuring cylinder, and then filter through a sterile filter unit with 2‐µm pore size (VWR 514‐0330). Dilute aliquots to 1× before use.

### Fluorescently labeled oligonucleotide (100 nM) (1 ml total volume)

Dry oligonucleotide with fluorescent dye modification (e.g., from Eurogentec)
RNase‐free water (Carl Roth, art. no. T143.5)

Store aliquots at −20°C. Avoid repeated freeze–thaw cycles (Davis, O'Brie, & Bentzley, 2000) and extensive exposure to light.

Dissolve the oligonucleotide delivered in dried form in RNase‐free water at a 100‐µM concentration. Dilute it to 100 nM, e.g., by adding 1 µl of oligonucleotide solution to 999 µl of RNase‐free water. Mix by pipetting up and down.

### Non‐stringent washing buffer, NSWB [6× SSPE, 0.01% (v/v) Tween 20] (1 L total volume)

300 ml of 20× SSPE (Sigma Aldrich S2015‐1 L)
1 ml of 10% Tween 20 (Sigma Aldrich 11332465001)
699 ml of Milli‐Q water (not DEPC‐treated)

Store protected from light at room temperature for up to 12 months. Mix the components in a measuring cylinder, and then filter through a sterile filter unit with 2‐µm pore size (VWR 514‐0334).

### NTP mix (2.5 mM each NTP)

NTP set (ThermoFisher Scientific, cat. no. R0481) with 100 mM each ATP, CTP, GTP, and UTP
RNase‐free water (Carl Roth, art. no. T143.5)

Store aliquots of the NTP mix at −20°C to avoid repeated freeze–thaw cycles. Mix equal volumes of all four NTPs, and then dilute to 1:10 with RNase‐free water.

### Primer oligonucleotide (1 µM) (100 µl total volume)

Purified oligonucleotide in dry form (e.g., from Eurogentec)
RNase‐free water (Carl Roth, art. no. T143.5)

Store aliquots at −20°C. Avoid repeated freeze–thaw cycles. (Davis et al., 2000). Dissolve the oligonucleotide delivered in dried form in RNase‐free water at a 100‐µM concentration. Further dilute it to 1 µM, e.g., by adding 1 µl of oligonucleotide solution to 99 µl of RNase‐free water.

### Stringent Washing Buffer SWB [101 mM of MES, 0.1 M Na^+^, 0.01% (v/v) Tween 20] (1 L total volume)

83.3 ml of 12× MES stock buffer
1 ml of 10% Tween 20 (Sigma Aldrich 11332465001)
5.2 ml of 5 M NaCl (Sigma Aldrich S5150)
910.5 ml of Milli‐Q water (not DEPC‐treated)

Store protected from light at 4°C for up to 12 months.

Mix the components in a measuring cylinder, and then filter through a sterile filter unit with 2‐µm pore size (VWR 514‐0334).

### Triton X‐100 0.25% (v/v) (1 ml total volume)

Triton X‐100 (Sigma Aldrich, cat. no. T8787)
RNase‐free water (Carl Roth, art. no. T143.5)

Store at 4°C for up to 12 months.

Prepare 1 ml of 0.25% (v/v) Triton X‐100 by adding 2.5 µl of Triton X‐100 to 997.5 µl of RNase‐free water in a sterile 1.5‐ml microcentrifuge tube and vortex.

## COMMENTARY

### Background Information

Oligonucleotide microarrays have proven very useful for screening interactions of biomolecules with nucleic acids. The commercial availability of these tools has greatly expanded our understanding of the interactions of DNA molecules, but the limitation of the technology to DNA has excluded other types of nucleic acids and modifications. Although it is well established that RNA is of high relevance in biological processes, comprehensive knowledge of its roles and interactions is still missing. RNA microarrays can be useful in elucidating the RNA interactome at a high‐throughput level that equally displays weak and strong binders, in strong contrast to other methods that select for the highest affinities only. However, the more delicate process of chemical oligonucleotide synthesis of RNA compared to DNA and the care in handling required due to the high sensitivity of RNA is primary responsible for the unavailability of RNA microarrays on a commercial scale.

In recent years, the interest in RNA has been driving the development of several approaches for the production of surface‐bound RNA libraries on an experimental scale. The most direct route is the chemical synthesis of RNA microarrays via photolithography (Lackey, Somoza, Mitra, Cerrina, & Damha, 2009; Lietard et al., 2018; Lietard, Damha, et al., 2019; Lietard, Schaudy, et al., 2019; Lietard & Somoza, 2019). It is a flexible method to generate RNA microarrays of very high densities, but with only short strands. A related method is based on the photolithographic synthesis of a microarray with both DNA and 2′*O*‐methyl RNA as a bases for the enzymatic conversion to an RNA microarray (Wu et al., 2014). However, the specialized instrumentation required for maskless array synthesis makes this method inaccessible to a broad audience. In a similar manner, reprogramming an Illumina sequencer in combination with *in vitro* transcription is also a feasible route toward surface‐immobilized RNA libraries of highest densities (Ozer et al., 2015), but its wider application is complicated by accessibility to this very specialized and expensive equipment. Transcription of spotted DNA allows for generation of very long RNA strands, but this comes at the cost of density due to the achievable size of the template DNA spots and an increase in the diameter of the spots upon transcription (Phillips et al., 2018).

In contrast to these existing approaches, this protocol describes the production of RNA microarrays based on manipulation of commercially available DNA microarrays with easily accessible tools and reagents, yielding an RNA microarray with resolution of individual spots and oligonucleotide lengths identical to those found on the template microarray. In the first steps of the protocol, hybridization of the primer specifically to its complementary sequence is essential to direct the position of photocrosslinking. Therefore, stringency washes are performed to remove the primer probe from sites with weaker base‐pairing interactions.

We have previously investigated different approaches for photocrosslinking (Hölz, Schaudy, Lietard, & Somoza, 2019) and thus decided to use C2 psoralen as a 5′‐modification on the primer probe (Pieles & Englisch, 1989). Psoralen derivatives have a long history in crosslinking of nucleic acids; after intercalation, preferentially at 5′‐TA‐3′ sites, exposure to 365‐nm UV light induces covalent bond formation with pyrimidine bases in a [2+2] cycloaddition reaction, in which only the di‐adduct yields an interstrand crosslink (Cimino, Gamper, Isaacs, & Hearst, 1985; Noll, Mason, & Miller, 2006). 5′‐C2‐psoralen‐modified oligonucleotides are commercially available, and the setup favors successful formation of the interstrand crosslink compared to the use of free psoralen. Furthermore, it allows for precise positioning of the crosslink to avoid interference with the enzymatic extension. We have observed an increased efficiency of the reaction for UV light exposure at 4°C compared to room temperature. After irradiation, a water wash ensures removal of primer that is not covalently bound.

Pre‐incubation prior to primer extension serves two purposes; the MES buffer used for crosslinking and hybridization contains EDTA, which complexes divalent cations. As these are crucial cofactors in a row of enzymatic reactions, the surface should be devoid of EDTA traces when starting the transcription. The MES buffer also contains sodium salts at high concentrations, which have been reported to inhibit T7 RNA polymerase activity (https://international.neb.com/faqs/2015/01/30/what‐are‐the‐main‐causes‐of‐reaction‐failure‐using‐t7‐rna‐polymerase1). To minimize the carry‐over of reagents from previous processing steps, the surface is pre‐conditioned by incubation in the transcription buffer before adding the primer extension reaction mixture.

Enzymatic extension of the crosslinked primer is performed by T7 RNA polymerase, which is widely used in *in vitro* transcription reactions because it very efficiently produces RNA from dsDNA templates containing a specific promoter sequence. In the absence of this sequence, a low yield of RNA has been reported (Krupp, 1988). Furthermore, examples for the transcription of short templates are reported with extended reaction times (Ghaem Maghami, Scheitl, & Höbartner, 2019; Lu & Li, 2012). In our hands, shorter incubation did not yield RNA in detectable quantities, and we therefore settled on running the reaction overnight.

The final step of the procedure involves enzymatic degradation of the DNA template strand, while keeping the single‐stranded RNA oligonucleotides attached to the surface. The setting of the primer extension in absence of a dsDNA template requires degradation of the DNA in a DNA:RNA hybrid. TURBO DNase and DNase I, in a less processive manner, have been reported as the enzymes of choice for removal of DNA from *in vitro* transcription reactions. However, in the classical *in vitro* transcription, 30 min of reaction time is recommended for the degradation of the dsDNA template, whereas the present setting requires substantially longer incubation with 5 hr for complete removal of the DNA strand. Furthermore, the degradation step substantially affects the yield of the process, as single‐stranded DNA linkers can also be substrates for the endonuclease. Therefore, the linker length is crucial. Shorter linkers are more stable toward nuclease degradation, but they also negatively affect the accessibility of the template strand for the polymerase in the primer extension. A detailed investigation of the effect of linker length on the process is described elsewhere (Schaudy et al., 2022). In our hands, the highest yield of conversion was achieved with a T_10_ linker on DNA microarrays from a commercial source. An alternative approach is to replace dT with dU in the DNA template strand, but not in the linker, enabling template‐specific degradation with uracil DNA glycosylase. Although this approach is effective (Hölz, Pavlic, Lietard, & Somoza, 2019), dU substitutions are generally not available in commercially available DNA microarrays, thus necessitating template degradation with TURBO DNase.

### Critical Parameters

#### Working RNase‐free

As RNA is susceptible to degradation by nucleases, the success of the conversion process crucially depends on an RNase‐free environment. To prevent contaminations, the operator should wear gloves at all stages of the protocol. Surfaces and instruments should be decontaminated with RNase AWAY surface decontaminant, and we recommend single‐use tubes and filter tips that are certified as RNase‐free. RNase‐free water should be used for preparing reaction mixtures, and buffers prepared in bulk should be filtered through a sterile filter unit and aliquoted in sterile reaction tubes upon use. RNase inhibitor is added to reaction mixtures in particularly sensitive processing steps. If the infrastructure allows, we recommend establishing a designated working place in the laboratory for experiments with RNA.

Support Protocol 2 describes an RNase H assay as a test to confirm the identity of the product of conversion. RNase H is known to degrade RNA exclusively in hybrids with DNA, while keeping single‐stranded RNA intact. To avoid contamination of other experiments, we recommend handling RNase H in a working place clearly separated from processing steps requiring an RNase‐free environment. Solutions containing RNase H should first be collected in a microcentrifuge tube, which is then thrown away, and buffers used for washing steps should be discarded immediately after use.

#### Multiple arrays per slide

If microarrays accommodating several individual arrays on a single slide are applied, e.g., a 4 × 44 K microarray, arrays currently not used should be covered with a hybridization chamber as a protection from mechanical damage (see Fig. 2B). To avoid carry‐over of reaction mixtures between arrays, the solutions should always be pipetted out of the hybridization chambers. Traces of the solution should be washed away by briefly pipetting buffer in and out of the hybridization chamber, before removing the chambers from all arrays on the surface to facilitate washing of the entire slide in buffers and drying it by centrifugation. Hybridization chambers applied only to cover a dry array can be reused after rinsing with RNase‐free water and drying with a stream of Argon.

#### Hybridization chamber

The dimensions of the hybridization chamber used may vary between different experiments, depending on the layout of the microarray. In our experience, reaction chambers should be filled to ∼80% of their total volume to keep an air bubble that facilitates mixing upon rotation. For incubation without rotation, e.g., during UV light exposure for crosslinking of the primer, and for washing steps, we recommend completely filling the reaction chamber. We have developed the current protocol with reaction chambers having volumes of ∼350 µl (Grace Bio‐Labs, type SA200) and ∼275 µl (type RD500958). For larger dimensions, potential effects on the efficiency of mixing by rotation and the homogeneous distribution of reagents should be taken into consideration.

#### Hybridization conditions

In general, the temperature for hybridization should be adjusted according to the characteristics (sequence, length, and chemistry) of the oligonucleotide probe. The conditions described in this protocol are verified for probes measuring 15 to 30 nt in length.

#### Characteristics of the primer

The sequence of the primer has been derived from previous work (Daube & von Hippel, 1992), and the 5′ terminus adapted for psoralen‐mediated photocrosslinking. Changes in the sequence and chemical modifications affect the stability and therefore might influence the efficiency of the conversion process. 2′*O*‐methyl RNA was chosen instead of RNA for the primer to ensure increased stability to nuclease degradation and base‐mediated hydrolysis.

#### Enzymatic reactions on the surface

As the oligonucleotides are bound to the solid support, the addition of reagents to stop enzymatic reactions is not required. Instead, solutions can be pipetted out of the reaction chamber, followed by a wash with buffer to remove traces of the enzyme on the surface.

#### Fluorescent dyes

Many fluorescent dyes are sensitive to light exposure. Reagents containing these dyes should be protected from light, for instance, by wrapping containers in aluminum foil and allowing solutions to thaw in the dark.

### Troubleshooting

Successful enzymatic conversion requires careful attention to the protocols for the three key steps: hybridization and photocrosslinking of the primer, primer extension, and template degradation. Particular attention should be paid to the critical parameters described above. In addition, Table 9 provides causes and solutions to common problems that may arise during enzymatic conversion experiments.

**Table 9:** Troubleshooting for Conversion of DNA to RNA Microarrays

Problem	Possible cause	Solution
No RNA detected.	Insufficient degradation of template DNA affects the accessibility of the RNA strand. Transcription reaction mixture has been prepared on ice. NTPs have degraded.	Verify the composition of the DNase reaction mixture and do not shorten incubation times. Prepare the solution for primer extension at room temperature. Use a fresh aliquot of NTPs and avoid repeated thawing.
Solution is leaking from the hybridization chamber.	The adhesive of the hybridization chamber is worn out. The adhesive seal tabs are not positioned properly.	Pipette out solutions, wash the slide briefly in RNase‐free water, dry, and use a new hybridization chamber. Carefully remove the adhesive seal tabs and replace with new ones.
Inhomogeneous fluorescent signal intensities across the microarray surface.	The bubble required in rotational mixing is trapped in one position instead of moving.	Before placing the slide in the hybridization oven, manually rotate it to verify movement of the bubble. If it is stuck, try to release it by carefully tapping the edges of the slide on the working bench.
Parts of the microarray are not converted.	The hybridization chamber was not correctly positioned.	A to‐scale drawing of the exact position of the hybridization chamber on the slide can be placed below the glass slide as a guide to properly align the hybridization chamber with the array.
Upon use of Cy3‐UTP, the background signal is very high.	Unspecific binding of Cy3‐UTP to the glass surface.	Wash the slide briefly in fresh FWB.
Low fluorescence signal.	Signal intensities of fluorescent dyes can depend on environmental parameters, for instance ionic strength or pH. Incorrect settings of microarray scanner.	Briefly wash the slide in a buffer with a composition corresponding to optimal light absorption, for instance with high pH in case of detection of fluorescein, before scanning. Tailor the wavelength and the emission filter of the microarray scanner to the excitation wavelength of the dye.
The adhesive seal tabs are not sticking well.	The surface of the hybridization chamber is wet, for instance, because some solution was pushed out upon applying it through the opposite hole.	Dry the surface with tissue paper. Pipette the solutions slowly and stop before applying air to the hybridization chamber. First seal the hole used to deliver the solution, and then the hole above the air bubble.

### Understanding Results

Completion of the Basic Protocol will yield a microarray with single‐stranded RNA. Fluorescent labeling allows for visualization of the success of the conversion process. This can be achieved, for instance, by following the Alternate Protocol to incorporate a nucleotide with fluorescent modification into the RNA product. This conversion in the presence of Cy3‐UTP allows detecting RNA directly after degradation of the DNA template. The example in Figure 5 shows that only DNA strands that allow for hybridization and crosslinking of the primer are converted into RNA, whereas features lacking the primer complement sequence stay dark. Fluorescent artifacts visible in the scan should be accounted for in data analysis by subtraction of the background signal.

As an alternative to internal labeling, hybridization with a fluorescently labeled complementary probe can detect the presence of specific ssRNA sequences after following the processing steps of the Basic Protocol, while keeping any non‐match RNA strands unaffected. The use of different probes in consecutive hybridization steps allows for the detection of different RNA sequences, as shown in Figure 6. Signal intensities are normalized to the average signal observed for the hybridization with probe X as the highest value. When comparing the results for hybridization to different RNA strands, the characteristics of the oligonucleotide (such as length, sequence, modifications, and sequence context of label) will impact the fluorescent signal intensities and the melting temperature, and these should thus be taken into consideration. Alignment of the scans with the design provided by the manufacturer helps identify the features on the surface targeted by the hybridization and extract the fluorescent intensity data for these control sequences implemented in the microarray design. Elevated hybridization signals for the expected sequences hint at a successful conversion to RNA, which can be further confirmed in an RNase H assay (Support Protocol 2) as a follow‐up experiment.

The data shown in Figure 7 are based on the conversion of two identical DNA arrays on a single slide according to the Basic Protocol, followed by hybridization with a Cy3‐labeled “template X” DNA probe, as described in Support Protocol 1. One array was treated with RNase H, and the other one was incubated only with the buffer as a control. Residual RNA was detected via hybridization with the Cy3‐labeled DNA probes “template X” and “template Y.” Signal intensities detected in the treated subarray were normalized to the signal in the control part, showing degradation (indicated as a major drop in fluorescence signal) only of the RNA in duplex with DNA, with no decrease in the signal intensities for hybridization with “template Y.” In addition to verifying that the product of the conversion is indeed RNA, these results also confirm that single‐stranded RNA stays intact in the presence of RNase H. However, another observation should be mentioned. The incubation in the absence of RNase H also induces loss of RNA from the surface during two consecutive hybridization steps to the same RNA sequence performed before (hybridization 1) and after (hybridization 2) incubation in the RNase H buffer, as shown in Figure 8. The effect on downstream experiments should therefore be taken into account.

### Time Considerations

The recommended incubation times are indicated at each step. Incubation times should be used to prepare reaction mixtures for the following processing step. The time required for scanning the slide depends on the resolution of the scan and the size of the array. Both the Basic Protocol and the Alternate Protocol can be accomplished in a day, considering the incubation for primer extension overnight. After generation of the RNA microarray, Support Protocol 1 can be completed in approximately 2 hr, and Support Protocol 2 in approximately 4.5 hr including scanning.

### Author Contributions

**Erika Schaudy**: conceptualization, data curation, investigation, methodology, validation, writing original draft, writing review and editing; **Jory Lietard**: funding acquisition, investigation, methodology, project administration, supervision, writing review and editing; **Mark M. Somoza**: conceptualization, data curation, funding acquisition, project administration, supervision, writing review and editing.

### Conflict of Interest

The authors declare no conflict of interest.

## Data Availability

The data are available from the corresponding author upon reasonable request.
